# Non-target toxicity of certain herbicides used in rice cultivation on mosquitoes

**DOI:** 10.1007/s10646-025-02880-1

**Published:** 2025-06-03

**Authors:** Aysegul Cengiz, Huseyin Cetin

**Affiliations:** Department of Biology, Faculty of Science, Akdeniz University, Antalya, 07070 Türkiye

**Keywords:** *Aedes*, *Culex*, Herbicides, Rice, Toxicity

## Abstract

This study aimed to investigate the non-target toxicity (insecticidal activity) of various herbicides (bentazone+MCPA, bispyribac sodium, cyhalofop butyl, quinclorac, oxadiazon, and clomazone) used in rice cultivation on *Aedes aegypti* L. and *Culex pipiens* L. mosquitoes. Given the ecological significance of rice cultivation areas (paddy fields) and the public health importance of these mosquito species, the research sought to understand how exposure to varying herbicide doses affects mosquito survival. The research utilized a laboratory strain of *Ae. aegypti* and both laboratory and field strains of *Cx. pipiens* mosquitoes, exposing them to three distinct doses of herbicides: the recommended application rate, and doses that were two and four times higher. Toxicity tests were conducted following the World Health Organization guidelines. Adult emergence inhibition rates (%) were recorded after exposing larvae of different developmental stages; early (first-second instar) and late (third-fourth instar) to the tested herbicides. Oxadiazon consistently showed higher toxicity to mosquitoes compared to other herbicides across all tested doses, causing up to 91.67% inhibition of adult emergence. Quinclorac also demonstrated notable toxicity, but to a lesser extent and primarily in *Ae. aegypti*. The remaining herbicides exhibited low or no significant insecticidal effects. Notably, early-stage larvae were more susceptible across the experiments. These findings suggest that while some herbicides used in paddy fields have a minimal impact on mosquitoes, oxadiazon, in particular, poses a significant risk. Understanding these interactions can aid in developing integrated pest management strategies that consider both crop protection and mosquito control, emphasizing the need for judicious herbicide use in ecologically sensitive environments.

## Introduction

Rice (*Oryza sativa* L.) (Poaceae), a semiaquatic monocotyledonous plant widely distributed around the world, requires significant amounts of water (Allard 1960; Kim et al. 2012). With the increase in the global population, the economic importance of rice has risen, supplying a substantial portion of the caloric intake for over 50% of the population (Ma et al. 2007; Fitzgerald et al. 2009). This plant is prevalent in countries with high rainfall and suitable temperatures, such as China, India, Indonesia, Bangladesh, and Vietnam. It can also be cultivated in many countries through irrigation from riverbeds and groundwater sources (Muthayya et al. 2014). Rice can complete its development in 15 cm of deep water at temperatures ranging from 20–25 °C within 90–180 days (Tasligil and Sahin 2011). It is noted that the optimal conditions range from 18–35 °C during germination and seedling stages, and 25–30 °C during seedling growth (Ogutcu et al. 1984).

In areas where rice is cultivated, herbicides are applied to the soil and water from the time of seed sowing until the harvest to combat competitive weeds that emerge during rice seed germination. The most commonly targeted weeds include the small-flowered nutsedge (*Cyperus difformis* L.), barnyard grass (*Echinochloa crus-galli* L.), European water-plantain (*Alisma plantago-aquatica* L.), sprangletop (*Leptochloa fascicularis* L.), yellowseed false pimpernel (*Lindernia dubia* L.), and early barnyard grass (*Echinochloa oryzoides* Ard.) (Sokat 2023). The herbicides used against these weeds fall into various categories, such as inhibitors of aceto-lactate synthase (ALS), photosynthesis, protoporphyrinogen oxidase (PPO), pigmentation, acetyl CoA carboxylase (ACCase), and cell wall synthesis (cellulose) (Nwani et al. 2010; Yang et al. 2010; He et al. 2014). The most frequently used active ingredients in rice cultivation areas are the herbicides bentazone+MCPA, bispyribac-sodium, cyhalofop-butyl, quinclorac, oxadiazon, and clomazone. To reduce economic losses, rice fields are extensively treated with these herbicides.

Rice production areas, being aquatic habitats, provide a home for numerous aquatic organisms, including a significant group; mosquitoes (Diptera: Culicidae). Mosquitoes, represented by over 3500 species globally, are prevalent in almost every region except the polar areas (Wilkerson et al. 2021). They are highly adaptable to various ecosystems and are known vectors of many diseases due to their blood-feeding behavior. Notable diseases transmitted by mosquitoes include Yellow Fever, Dengue Fever, West Nile Fever, and Malaria (Brugueras et al. 2020). The water in rice fields offers an optimal environment for mosquito development due to its suitable temperature and depth. Consequently, these fields serve as breeding and developmental habitats for several mosquito species known to be vectors of various diseases (Zhao and Xue 2022; Kaboré et al. 2023). It is hypothesized that pesticides, particularly herbicides used in rice cultivation, could influence mosquito development, potentially impacting mosquito populations either positively or negatively.

This study investigated the toxicity of herbicides predominantly used in rice fields such as bentazone+MCPA, bispyribac sodium, cyhalofop butyl, quinclorac, oxadiazon, and clomazone on early (first-second) and late (third-fourth) instar larvae of *Culex pipiens* L. and *Aedes aegypti* L. mosquitoes to determine if different ages exposed to various doses could successfully mature into adults.

## Materials and methods

### Tested mosquitoes

In this study, two mosquito species were used: *Ae. aegypti* (Bora Bora strain) and *Cx. pipiens* laboratory and field strains. The *Ae. aegypti* Bora Bora strain was provided by Dr. Oner Kocak from Hacettepe University, Ankara, Türkiye. The *Cx. pipiens* laboratory strain was originally collected from Arapsuyu, Antalya in 2007 and has been maintained in continuous colony culture at the Vector Ecology and Control Laboratory, Department of Biology, Akdeniz University, Antalya under controlled conditions (24 ± 2°C, 60 ± 10% relative humidity, (12:12) light-dark photoperiod). The *Cx. pipiens* field strain was collected from a pool located within Akdeniz University campus, and the colony was established in the laboratory with individuals reared for several generations before experiments were conducted. The development of these cultured mosquitoes necessitated blood, fish food, sugary water, and amino acid solutions, all of which were provided in the laboratory.

### Toxicity tests

Tests were conducted following a larvicide test method modified from the World Health Organization guidelines (WHO 2005). The herbicides utilized in these tests have been approved for field application by the Ministry of Agriculture and Forestry of the Republic of Türkiye and were employed at their label dose, as well as at two and four times this dose. These herbicides were identified after discussions with farmers and pesticide applicators in the Edirne province, a prominent area for rice production in Türkiye (Table 1). The tests were carried out in plastic containers with a surface area of 171 cm², each containing 500 mL of settled tap water. Herbicides were diluted with water to achieve the desired dose and mixed into the test environment using a spatula for 10 s. In the tests, 10 mosquito larvae at either early (first-second) or late (third-fourth) stages were used per replicate, with the tests conducted four times in total.

**Table 1:** Overview of tested herbicides and application doses

Herbicides	Product name	Rates (%) in formulations and type of formulation	Application doses (ml formulation/da)		
			RD	2xRD	4xRD
Bentazone+MCPA	Aquadex	%25 SL	200	400	800
Bisyribac sodium	Dalia 400 SC	%42 SC	5	10	20
Chalofop butyl	Fedora	%20 EC	75	15	300
Quinclorac	Otör IQ	%25 OD	200	400	800
Oxadiazon	Dinox CS	%20 CS	150	300	600
Clomazone	Titan 48 EC	%48 EC	70	140	280

*RD* recommended dose, *SC* suspension concentrate, *EC* emulsifiable concentrate, *OD* oil dispersion, *CS* capsule suspension, *SL* soluble liquid

**Fig. 1:**
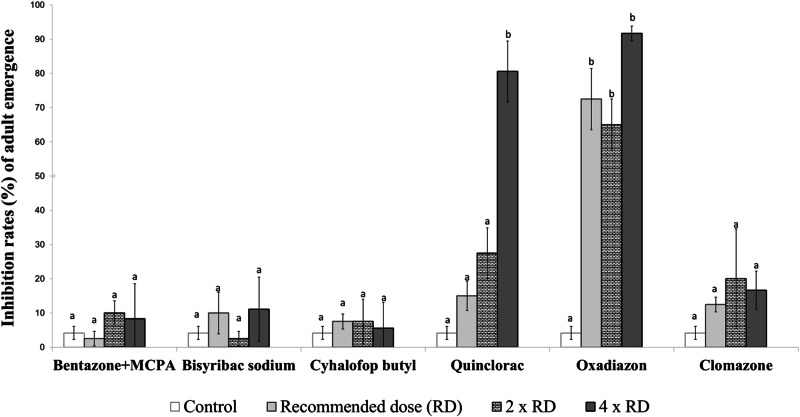
Inhibition rates (%) of adult emergence from early-stage larvae of the *Aedes aegypti* laboratory strain exposed to herbicides. Different lowercase letters on the bars indicate statistically significant differences between tested herbicides within the same dose (Duncan Multiple Range Test, *p* ≤ 0.05)

**Fig. 2:**
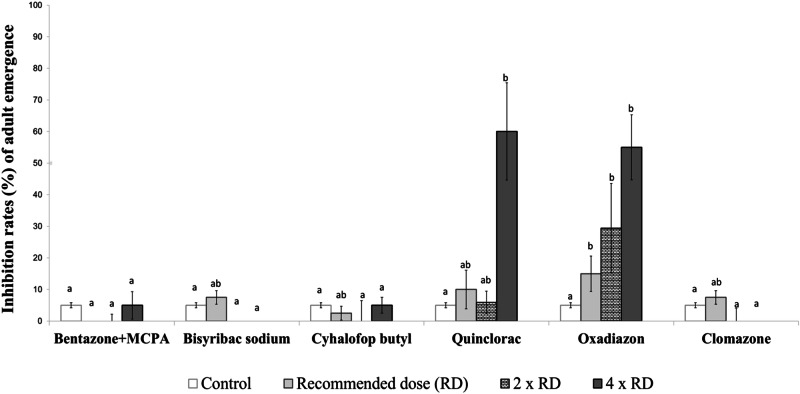
Inhibition rates (%) of adult emergence from late-stage larvae of the *Aedes aegypti* laboratory strain exposed to herbicides. Different lowercase letters on the bars indicate statistically significant differences between tested herbicides within the same dose (Duncan Multiple Range Test, *p* ≤ 0.05)

**Fig. 3:**
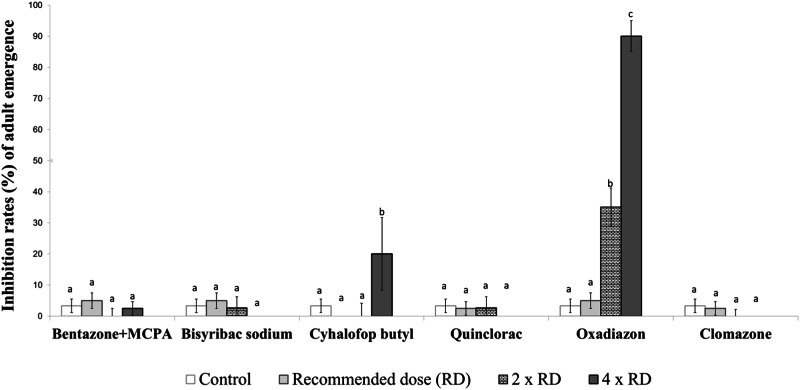
Inhibition rates (%) of adult emergence from early-stage larvae of the *Culex pipiens* field strain exposed to herbicides. Different lowercase letters on the bars indicate statistically significant differences between tested herbicides within the same dose (Duncan Multiple Range Test, *p* ≤ 0.05)

**Fig. 4:**
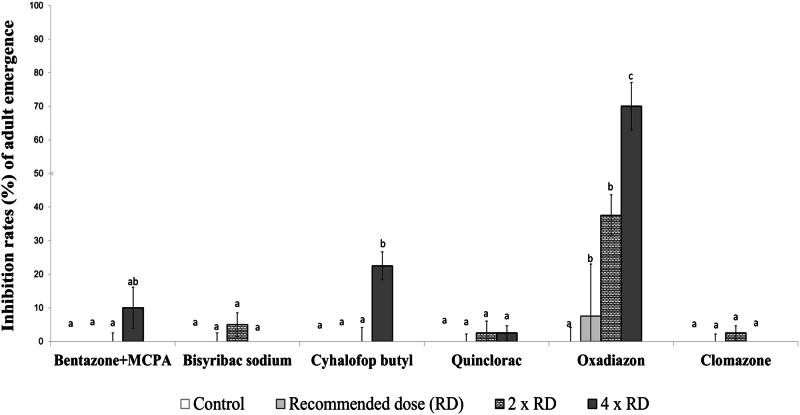
Inhibition rates (%) of adult emergence from late-stage larvae of the *Culex pipiens* field strain exposed to herbicides. Different lowercase letters on the bars indicate statistically significant differences between tested herbicides within the same dose (Duncan Multiple Range Test, *p* ≤ 0.05)

**Fig. 5:**
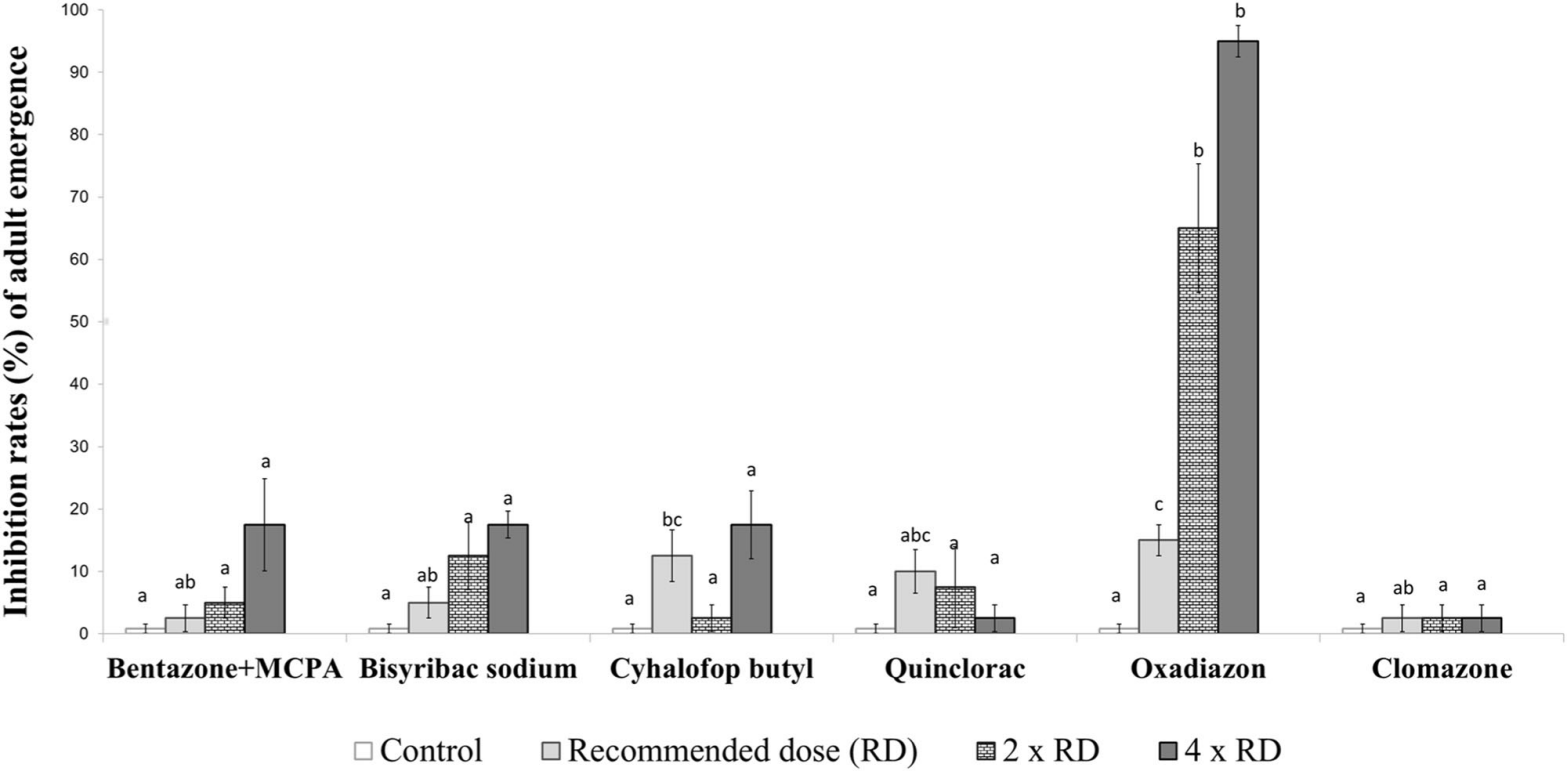
Inhibition rates (%) of adult emergence from early-stage larvae of the *Culex pipiens* laboratory strain exposed to herbicides. Different lowercase letters on the bars indicate statistically significant differences between tested herbicides within the same dose (Duncan Multiple Range Test, *p* ≤ 0.05)

**Fig. 6:**
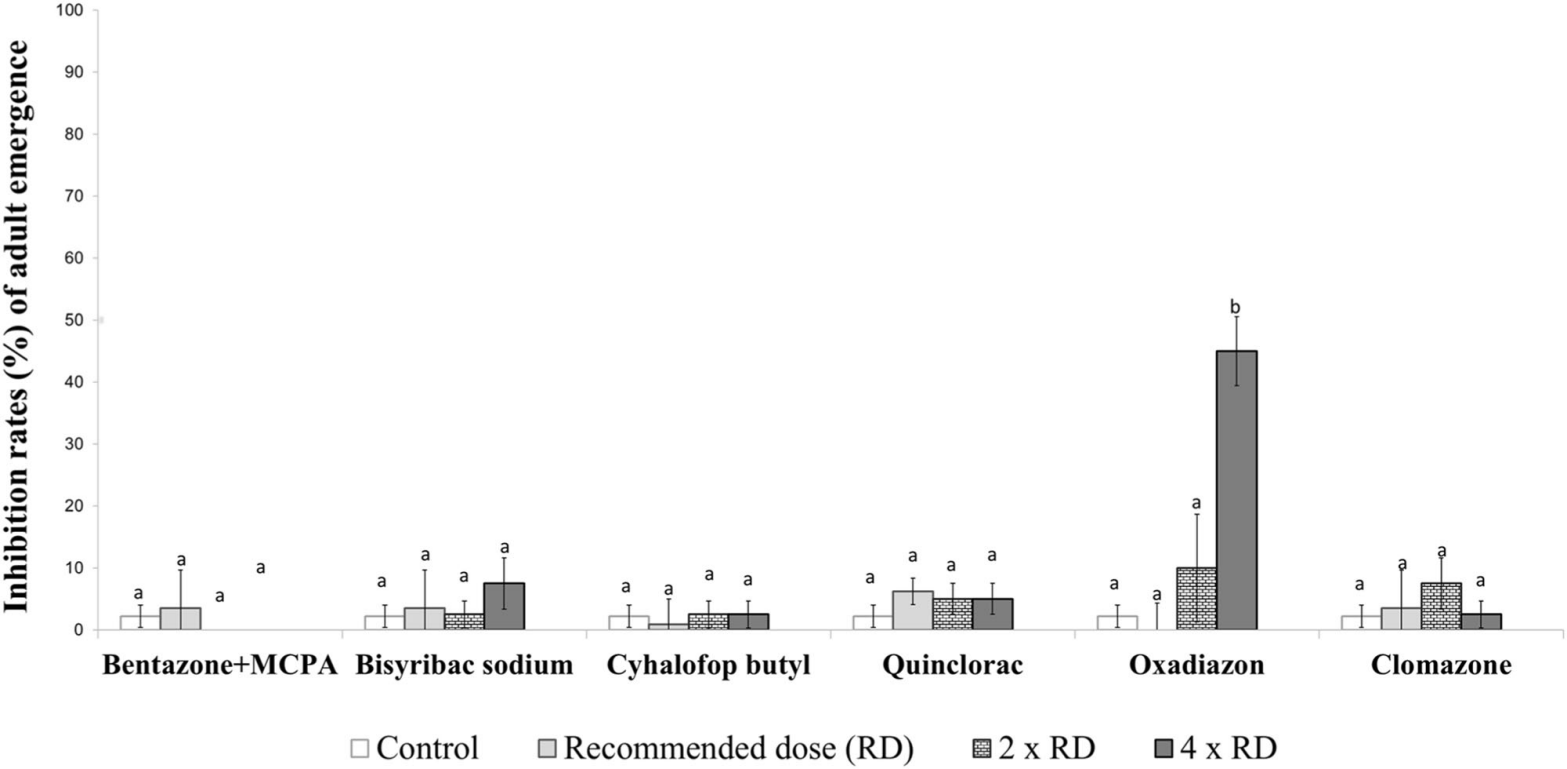
Inhibition rates (%) of adult emergence from late-stage larvae of the *Culex pipiens* laboratory strain exposed to herbicides. Different lowercase letters on the bars indicate statistically significant differences between tested herbicides within the same dose (Duncan Multiple Range Test, *p* ≤ 0.05)

The larvae exposed to the herbicides were monitored until all had either emerged as adults or failed to do so. The adult emergence inhibition rates (%) were then calculated as the proportion of larvae that did not successfully emerge as adults by the end of the experiment. In this study, adult emergence inhibition rates refer specifically to the failure of larvae to reach adulthood, rather than direct mortality at earlier larval or pupal stages. We did not separately record deaths occurring at the larval or pupal stages. Instead, we continued observations until the last individual had either successfully emerged as an adult or failed to do so, at which point the final adult emergence inhibition rate was determined (Figs. 1–6). The duration of the bioassay was set to a minimum of 14 days for all test groups, ensuring that both early-stage (first-second instar) and late-stage (third-fourth instar) larvae had sufficient time to either successfully emerge as adults or fail to do so. This extended observation period allowed us to assess chronic toxicity effects rather than only acute toxicity that could occur within 24–48 h.

### Statistical analysis

The percent adult emergence inhibition rates of mosquitoes were assessed for statistical comparisons within each herbicide, comparing the effects of different doses, including the control group. Statistical analyses were conducted using the Duncan Multiple Range Test at a *p* ≤ 0.05 significance level in the SPSS statistical analysis software (Version 20.0). If the mortality rate in the control group ranged from 5–20%, the observed mortality rate was adjusted using (Abbott’s 1925) formula. Should the mortality rate in the control group exceed 20%, the tests were deemed invalid and subsequently repeated.

## Results

The toxicity of six herbicides on early-stage larvae of the *Ae. aegypti* laboratory strain were examined across three doses. According to the results, oxadiazon exhibited a higher toxicity on the early-stage mosquito larvae at all tested doses compared to the other herbicides. Oxadiazon caused adult emergence inhibition rates ranging from 65–91.67% at the tested doses. Following oxadiazon in terms of insecticidal activity was quinclorac, which resulted in adult emergence inhibition rates between 15 and 80.56% in early-stage larvae. No significant adult emergence inhibition rates were detected in the early-stage larvae with the other herbicides when compared to the control group (Fig. 1).

When comparing the toxicity of herbicides on the late-stage larvae of *Ae. aegypti* at the tested doses, it was again observed that oxadiazon and quinclorac exhibited high insecticidal activity. In contrast, the other herbicides did not show a significant insecticidal effect when compared with the control group. At this stage, oxadiazon caused adult emergence inhibition rates ranging from 15–55%, while quinclorac resulted in adult emergence inhibition rates between 10 and 60% (Fig. 2).

When the toxicity of the tested herbicides on the early-stage larvae of the *Cx. pipiens* field strain was examined, oxadiazon was notably the most effective, causing adult emergence inhibition rates ranging from 35.1–90%. Among the other tested herbicides, only cyhalofop butyl at four times the recommended dose for rice cultivation caused statistically significant and higher adult emergence inhibition rates (20%) compared to the control group. Bentazone+MCPA, bispyribac sodium, quinclorac, and clomazone did not result in statistically significant adult emergence inhibition rates when compared with the control group (Fig. 3).

When the insecticidal effects of herbicides on the late-stage larvae of *Cx. pipiens* were examined, oxadiazon stood out significantly, with its lethal effectiveness increasing from 37.5–70% as the dose used was escalated. In terms of insecticidal activity, only cyhalofop butyl at the highest dose followed oxadiazon, causing 20% mortality. The other herbicides did not exhibit significant insecticidal activity when compared with the control group (Fig. 4).

When the toxicity of the tested herbicides on the early-stage larvae of the *Cx. pipiens* laboratory strain was examined, only oxadiazon was found to cause adult emergence inhibition rates ranging from 65–95%. The other five herbicides did not exhibit significant insecticidal activity when compared with the control group (Fig. 5).

Similar to its effect on early-stage larvae, in the late-stage larvae of the *Cx. pipiens* laboratory strain, only oxadiazon caused adult emergence inhibition rates ranging from 10–45%. When compared with the control group, the adult emergence inhibition rates from the other herbicides did not show any statistically significant difference in toxicity (Fig. 6).

When comparing the responses of laboratory and field strains of *Cx. pipiens* to the tested herbicides, no statistically significant differences were observed in most cases. However, oxadiazon exhibited slightly lower toxicity in field strain compared to laboratory strain, which may suggest some level of tolerance due to prior environmental exposure. Nevertheless, this difference was not statistically significant (*p* > 0.05). For the other tested herbicides, both strains exhibited similar susceptibility levels. These findings indicate that field populations have not developed a strong tolerance to the tested herbicides at the doses used in this study, although further investigations involving long-term exposure studies would be necessary to confirm this hypothesis.

## Discussion

The necessity to meet the world’s food needs is becoming increasingly challenging due to factors such as rising industrialization, haphazard urbanization, and the degradation of agricultural lands. To cope with these challenges and enhance the quality of agricultural produce, farmers are resorting to the use of pesticides. Globally, over two million tons of pesticides are applied annually to protect public health, agriculture, forests, and veterinary fields, with this amount reaching 53,672 tons in Türkiye in 2020 (Ozercan and Tascı 2022).

Pesticides, derived from chemical or biological sources, are employed to eliminate target pests. However, their overuse and indiscriminate application can result in contamination of soil and water, leaving residues and causing adverse effects on non-target organisms, including mammals, birds, and fish (Ankit et al. 2020).

Rice fields provide a suitable habitat for various organisms, especially mosquitoes, due to the temperature, water amount, and topographic features required by the crop (Ohba et al. 2013; Kasamesiri and Thaimuangphol 2015). The persistence of stagnant water in the fields and occasional replacement with fresh water facilitate the population dynamics of mosquito larvae. As a result, in agricultural fields, chemical and biological control methods are employed together to combat these pests. Research indicates that mosquitoes have developed resistance to many insecticides used in chemical control (Liu 2015; Ser and Cetin 2019). Furthermore, the application of herbicides in rice fields to manage weed populations can directly impact the environment where mosquitoes reside, indirectly contributing to contamination at different stages.

This research evaluated the toxicity of six herbicides—bentazone+MCPA, bispyribac sodium, cyhalofop butyl, quinclorac, oxadiazon, and clomazone—utilized in rice farming on the larvae of *Ae. aegypti* and *Cx. pipiens* mosquitoes. The findings underscored that among these herbicides, oxadiazon demonstrated the most significant toxic impact on the larvae of both mosquito species. In general, *Aedes* larvae showed greater sensitivity than *Culex* larvae throughout the toxicity tests, with early-stage larvae exhibiting greater susceptibility than late-stage larvae in all experiments.

Our study revealed that the tested herbicides variably affected mosquito larvae, with oxadiazon standing out for its strong toxicity at all doses and strains, resulting in adult emergence inhibition rates exceeding 90% for both mosquito species. In contrast, quinclorac exhibited toxicity only against *Aedes* larvae, and only at the highest dose applied. Except for oxadiazon and quinclorac, the other four herbicides appear to have low toxicity in all strains.

The potential role of cross-resistance between herbicides and insecticides should also be considered when interpreting these findings. While our study did not specifically test mosquito susceptibility to insecticides, previous research suggests that metabolic resistance mechanisms, such as cytochrome P450 monooxygenases, glutathione S-transferases, and esterases, may contribute to tolerance against multiple chemical classes, including both insecticides and herbicides (Liu 2015; Ser and Cetin, 2019). Future studies should explore whether mosquitoes that exhibited lower susceptibility to certain herbicides have developed cross-resistance due to previous exposure to insecticides or if they possess independent mechanisms to metabolize herbicides.

It is thought that differences in the susceptibility potential of strains to herbicides may be due to detoxification enzymes and systems in the larvae’s bodies. Detoxification enzymes such as cytochrome P450, microsomal oxidases, glutathione S-transferases, and hydrolases are responsible for the metabolism of pesticides and the development of resistance (Agosin 1985; Ju 2004; Londoño et al. 2004). In this context, future studies should investigate detoxification mechanisms and enzyme levels in mosquitoes and non-target species exposed to different herbicides.

Literature encompasses studies by diverse researchers regarding the toxicity of herbicides to non-target organisms. In research conducted by (Fathy et al. 2021), the toxic impacts of bispyribac sodium, penoxsulam, haloxyfop-p-ethyl, pinoxaden, and tralkoxydim herbicides, as well as the pyrethroid insecticide deltamethrin, were assessed on the late larval stages and pupae of *Cx. pipiens* over 24, 48, and 72 h exposure periods. The findings revealed that deltamethrin exhibited the highest insecticidal activity among the substances tested, with pinoxaden being the most potent herbicide at impeding both developmental stages. Bispyribac sodium and penoxsulam showed moderate insecticidal activity levels, whereas the remaining herbicides demonstrated minimal toxic effects. Furthermore, an increasing trend in toxicity against both mosquito larvae and pupae was observed over time, notably after 72 h for larvae and 48 h for pupae.

In a study by (Morris et al. 2016) the toxicological impacts of two commercially prevalent herbicides in rice cultivation—Beyond (containing imazamox) and Roundup (containing glyphosate)—on *Ae. aegypti* larval development were investigated. The research tested various concentrations (0.74, 1.49, 2.24 μL/m^2^ for imazamox and 270, 550, 820 μg/m^2^ for glyphosate) and found distinct effects on mosquito larvae. Roundup did not significantly impact mosquito survival or development at low and medium doses, but its highest concentration delayed the time to adult eclosion. On the other hand, Beyond was found to enhance survival rates at lower concentrations, although it decreased female mass at the highest tested concentration.

In the research conducted by (Nikbakhtzadeh and Fuentes 2022), the study examined how glyphosate influences the oviposition behavior and larval development in *Culex quinquefasciatus*. The findings revealed that glyphosate concentrations of 1000 ppm were fatal to the larvae, obstructing their molting process and thus inhibiting their progression to the pupal stage or adulthood. Similarly, exposure to 500 ppm glyphosate significantly extended the larval period and postponed the transition to the pupal phase. At both concentration levels, total larval mortality was observed.

(Baglan et al. 2018) explored the impact of glyphosate—a herbicide with widespread global usage—on the learning behaviors of *Ae. aegypti* larvae. The study assessed the larvae’s escape behaviors and speeds at glyphosate concentrations of 50, 100, 210 µg/L, and 2 mg/L. The results demonstrated a concentration-dependent impairment in the larvae’s learning abilities, with increasing concentrations of glyphosate correlating with slower escape responses.

Additionally, (Talib et al. 2018) investigated the acute effects of Roundup Ultra, a herbicide with glyphosate as its active ingredient, on *Gambusia affinis* over a 72 h period at varying concentrations (5, 8, 11, 14, 17, 20, 23, 26, 29, 32 mg/L). They established an LC_50_ value of 17.82 mg/L and observed that mortality rates increased with the herbicide concentration. Notably, the fish exhibited behavioral changes at elevated concentrations, including irregular swimming patterns, hyperactivity, loss of equilibrium, enhanced operculum movement, and more frequent contact with the sides of the aquarium.

(Nakagome et al. 2006) performed a deterministic risk analysis on a range of herbicides frequently utilized in rice cultivation, namely oxyfluorfen, oxadiazon, clomazone, 2,4-D, bispyribac-sodium, methyl metsulfuron, ethyl carbofentrazone, quinclorac, ethyl pyrazosulfuron, and bentazone—to evaluate their effects on *Daphnia magna*, an organism that serves as a food source for mosquito larvae. This 48 h study aimed to ascertain their EC_50_ values. The findings highlighted that the herbicides oxyfluorfen and oxadiazon were significantly more toxic to *D. magna*, with respective EC_50_ values of 3.18 and 3.54 mg/L. The study concluded that the other tested herbicides exerted minimal or negligible impact on *D. magna*.

In a study examining the impact of herbicides on aquatic life, the 96 h LC_50_ values for clomazone, metsulfuron-methyl, and quinclorac-herbicides frequently used in rice farming-were determined for the silver catfish *Rhamdia quelen*. The LC_50_ was established at 7.32 µL/L for clomazone and 395 mg/L for quinclorac. Remarkably, for metsulfuron-methyl, no LC_50_ value could be identified as all fry survived even at a high concentration of 1200 mg/L. This outcome suggests that among the tested substances, only clomazone poses a significant mortality risk to cultured silver catfish (Miron et al. 2004).

Evaluation of existing literature corroborates the findings of this study, indicating that herbicides utilized in rice paddies exhibit varying degrees of toxicity to mosquito larvae, ranging from low to high. Specifically, the active ingredient oxadiazon was identified as causing significant mortality among larvae and exhibiting toxic effects on non-target species. These observations underscore the need for additional research to ascertain the viability of using oxadiazon as a larvicide in mosquito management strategies and to elucidate the mechanisms underlying its insecticidal activity.

Our results underscore the necessity of accounting for the use and prevalence of various pesticide groups in mosquito control strategies. Research aimed at mitigating mosquito resistance to insecticides indicates that overlooking herbicides’ impacts might enhance control efforts’ efficacy. Therefore, it is crucial for practitioners to carefully monitor herbicide dosages to minimize environmental residues and potential toxicity. Moreover, when deploying these chemicals, it is essential to consider the possibility of cross-resistance development in insects to different pesticide categories, ensuring that applications are strategic and informed.

Mitigating excessive insecticide use in chemical control is crucial for economic benefits, public health, and preserving ecological balance. Consequently, in the context of herbicide applications aimed at enhancing agricultural quality and productivity, it is imperative to comprehensively assess potential ecological risks. Specifically, the unregulated application of potent chemicals, such as oxadiazon highlighted in our research, must be recognized for its potential to provoke environmental challenges.

Ultimately, effective control measures necessitate a comprehensive focus that extends beyond isolated interventions. Collaboration between researchers and practitioners is essential to safeguard and sustain ecological equilibrium. Through such cooperative efforts, we can adopt strategies that ensure the enduring vitality of our planet, securing a resilient and sustainable environment for future generations.

## Data Availability

Data will be available under request to the corresponding author.
